# Post-silking carbon partitioning under nitrogen deficiency revealed sink limitation of grain yield in maize

**DOI:** 10.1093/jxb/erx496

**Published:** 2018-01-18

**Authors:** Peng Ning, Lu Yang, Chunjian Li, Felix B Fritschi

**Affiliations:** 1 Key Laboratory of Plant-Soil Interactions, Ministry of Education, Department of Plant Nutrition, China Agricultural University, Beijing, China; 2 College of Natural Resources and Environment, Northwest A&F University, Yangling, Shaanxi, China; 3 Division of Plant Sciences, University of Missouri, Columbia, USA; 4 Institute of Agricultural Resources and Regional Planning, Chinese Academy of Agricultural Sciences, Beijing, China

**Keywords:** Carbon, nitrogen, post-silking partitioning, source–sink relationship, sugar transport, yield, *Zea mays*

## Abstract

Maize (*Zea mays*) plants exhibit altered carbon partitioning under nitrogen (N) deficiency, but the mechanisms by which N availability affects sugar export out of leaves and transport into developing ears remain unclear. Maize was grown under field conditions with different N supply. Plant growth, sugar movement, and starch turnover in source or sink tissues were investigated at silking and 20 or 21 days after silking. Nitrogen deficiency stunted plant growth and grain yield compared with N-sufficient plants, and resulted in greater starch concentrations in leaves due to more as well as larger starch granules in bundle sheath cells. Transmission electron microscopy revealed an open symplastic pathway for sucrose movement in N-deficient leaves, while the expression levels of transporters responsible for sucrose efflux and phloem loading were lower than in N-sufficient leaves. Nonetheless, greater starch concentrations in the apical cob portion of N-deficient plants implied sufficient carbon supply relative to the diminished sink strength (decreased kernel number and weight). Together with the high sugar concentrations in the developing kernels, the results indicated that reduced sink capacity and sugar utilization during grain filling may limit the yield in N-deficient plants, which in turn imposes a feedback inhibition on sugar export from leaves.

## Teaser

Anatomical, physiological, and molecular analysis revealed that yield losses in nitrogen-deficient maize were more closely associated with limited capacity of sugar utilization in developing ears than sucrose export from leaves.

## Introduction

In maize (*Zea mays*), approximately half of the total dry matter at maturity is accumulated between planting and silking and the other half during the reproductive period (Lee and Tollenaar, 2007; Ning *et al.*, 2013). At the whole plant level, improvement of crop yield may be obtained through increasing carbohydrate production in source leaves (Wang *et al.*, 2015) and/or enhancing the utilization of photoassimilates in sink organs (Sosso *et al.*, 2015). Nitrogen (N) plays a fundamental role in both source and sink establishment (Hawkesford *et al.*, 2012). To meet the high N demand of grain filling, large amounts of N are progressively remobilized from vegetative organs during reproductive growth (Masclaux-Daubresse *et al.*, 2010). For N-deficient plants, this process is particularly pronounced and is reflected in accelerated leaf senescence. As such, leaf N is a pivotal factor influencing photosynthesis and carbon (C) production after silking, and an important source of N for grain filling. Interestingly, many studies have shown that more starch is accumulated in source leaves under N-deficient than N-sufficient conditions in maize (Peng *et al.*, 2013, 2014) as well as in other plants (Scheible *et al.*, 1997; Wang *et al.*, 2004; Zhao *et al.*, 2015). These studies clearly indicate that leaf N status impacts C partitioning and mobilization from leaves. Thus, both C capture and its export from source leaves are critical for yield formation under N deficiency.

Generally, N starvation leads to lower sink demand for photoassimilates, resulting in a feedback regulation on the storage of accumulating sugars as transitory starch in source tissues (Jeannette *et al.*, 1995). In turn, N addition stimulates sugar and starch partitioning into organic acid pools to support amino acid biosynthesis and assimilation (Scheible *et al.*, 1997). Clearly, N deficiency can decrease C assimilation, while C starvation reduces N utilization in plants (Cazetta *et al.*, 1999). Based on the hyper-accumulation of starch in N-deficient maize leaves, Peng *et al.* (2014) speculated that photosynthate availability *per se* may not be the main factor responsible for yield reductions under N-deficient conditions; rather impaired sugar mobilization and translocation out of leaves likely contributes to yield reduction. Mutations blocking either starch synthesis or starch breakdown have been reported to reduce plant growth in Arabidopsis, maize, and other species (Schlosser *et al.*, 2012; Stitt and Zeeman, 2012). Starch degradation could be accelerated on warm nights to meet the increased demand for maintenance and growth (Pilkington *et al.*, 2015). Collectively, these results highlight the importance of sugar export and starch turnover in plant growth and resilience to stress (Sulpice *et al.*, 2009; Pilkington *et al.*, 2015).

Sucrose is a principal form of photoassimilate in the long distance transport from source to sink in maize. Initially, in the leaves, sucrose diffuses through plasmodesmata from mesophyll cells (MC) to bundle sheath (BS) and then to vascular parenchyma (VP) cells (Braun and Slewinski, 2009). The last steps of sucrose movement to the phloem in maize source leaves involve an apoplastic strategy (Braun and Slewinski, 2009), where sucrose is exported from VP cells to the apoplast by SWEETs (sucrose will eventually be exported transporters) (Chen *et al.*, 2012), and then retrieved from the apoplast and loaded into phloem companion cells by sucrose transporters (SUTs) (Slewinski *et al.*, 2009; Baker *et al.*, 2016). Once in the phloem, long distance transport can be impaired at different steps by a number of factors, including callose deposition (Russin *et al.*, 1996; Ma *et al.*, 2008). Interestingly, in wheat (*Triticum aestivum*), yield reduction in N-deficient plants was at least partially attributed to increasing callose deposition in peduncle vascular bundles during middle grain-filling (Kong *et al.*, 2013).

Following loading and transport in the phloem, sucrose arrives in the sink tissues. In reproductive maize, it arrives at the cob, which serves as a transient storage for assimilates, prior to uptake by kernels. Sucrose is largely cleaved by vacuolar or cell wall invertases into fructose and glucose in the maternal tissues, such as the pedicel, the placenta–chalaza, and especially by cell wall invertases in the basal endosperm transfer layer (Shannon, 1972; Bihmidine *et al.*, 2013). The resulting hexoses are then transferred into the endosperm through an apoplastic pathway via transporters and effluxers, and utilized for the synthesis of starch and other end products (Shannon, 1972; Bihmidine *et al.*, 2013). The sugars also can be stored locally as transient starch in the lower pericarp and pedicel (Mäkelä *et al.*, 2005; Bihmidine *et al.*, 2013). Overall, these results emphasize the prominent role of carbohydrate interconversion and transport between the maternal tissues and developing kernels, but the influence of N supply on these processes is not fully understood in maize.

Maize plants exhibit plasticity and adaption to N deficiency, such as favoring of sugar partitioning towards starch (Peng *et al.*, 2013, 2014) or cell wall cellulose (Schlüter *et al.*, 2012) in leaves, in turn allowing photosynthesis to proceed. While starch *per se* is a storage carbohydrate without demonstrated regulatory activities, it has been implicated as a major integrator in the regulation of plant growth (Sulpice *et al.*, 2009). Although much research has been conducted into C and N metabolism in maize, the mechanisms underlying and implications associated with starch built up in leaves of N-deficient maize are not understood. The objective of this research was to investigate how N availability affects sugar export from leaves and movement to developing ears in field-grown maize.

## Material and methods

### Experimental design and field conditions

Field experiments were conducted in 2014 and 2015 at the University of Missouri Bradford Research Center near Columbia, MO, USA (38°53′ N; 92°12′ W) on a Mexico silt loam (fine, semectitic, mesic vertic epiaqualfs) soil (CARES, 2012). The chemical properties of the top 20 cm soil layer sampled prior to planting in 2014 and 2015 were, respectively, pH 6.3 and 6.5, organic matter 2.8 and 3.1%, N_min_ (NO_3_^−^+NH_4_^+^) 12.9 and 11.8 mg kg^−1^, Bray-I P 14.0 and 6.4 mg kg^−1^, and NH_4_OAc-K 78.0 and 78.1 mg kg^−1^.

Previous studies revealed that plant growth and carbon partitioning in maize hybrid ‘Pioneer 32D79’ are highly responsive to soil N availability in both greenhouse and field conditions (Peng *et al.*, 2013, 2014, 2016). Thus, this hybrid was selected for the present study and was sown on 6 May 2014 and 8 May 2015 to achieve a stand density of 78 000 plants ha^−1^. Nitrogen deficiency resulted in approximately 1 d delay in silking and 7–9 d earlier maturity than sufficient N supply. Plots were 12.2 × 6.1 and 6.1 × 6.1 m^2^ in 2014 and 2015, respectively. Weeds were controlled by pre-emergence herbicide application (Atrazine plus S-metolachlor) followed by manually hoeing as needed. Three N treatments was used consisting of (i) N_0_, no N fertilizer as control; (ii) N_200_, 200 kg N ha^−1^ split equally at emergence and V8 stages (eighth leaf with visible ligule); and (iii) N_300_, 200 kg N applied as described for the N_200_ treatment, and an additional 100 kg N ha^−1^ applied 1 week before silking. The three N treatments were imposed in a randomized complete block design with four replications. Nitrogen was applied manually in the form of urea. The amount of precipitation during the maize growing season in May, June, July, August, and September was, respectively, 70, 164, 41, 57, and 186 mm in 2014 and 140, 129, 204, 106, and 21 mm in 2015.

### Plant sampling and measurements

Maize above-ground parts were harvested at silking, at 21 (in 2014) or 20 (in 2015) days after silking (DAS), and at physiological maturity. The whole-plant samples from four visually uniform plants were cut at the stem base in each plot between 09.00 and 11.00 h at each sampling. The shoot was divided into ear-leaves, other leaves, stems, and ear, and the ear was further divided into apical and basal halves. The number of ovaries or kernels was counted and they were separated from the cob. Tissues were dried at 60 °C to a constant weight, weighed, and ground to pass a 1 mm screen. Subsamples were further processed to fine powder with a Geno/Grinder 2010 (SPEX SamplePrep, Metuchen, NJ, USA). Carbon and N concentrations were determined with an Elementar vario Macro Cube CHNS analyser (Germany) with phenylalanine as a standard. The maximum area of the ear leaves was measured at silking using a leaf area meter (LI-3100, LI-COR, Inc. Lincoln, NE, USA).

In 2014, separate ear-leaf samples for carbohydrate analysis were collected between 09.00 and 11.00 h in the morning at silking and 21 DAS. Additionally, leaf disks (2 mm diameter) for transmission electron microscopy (TEM) were cut from the longitudinal middle of the leaf blade and immediately transferred to fixative (2% paraformaldehyde, 2% glutaraldehyde in 100 mM sodium cacodylate buffer pH 7.35). In 2015, ear-leaf samples were collected every 4 h starting from 08.00 h at silking and 20 DAS. The middle (~10 cm) of the leaf blade was dissected to remove the midrib and the leaf blade tissues from either sides of the midrib were processed for gene expression or carbohydrate analyses. The leaf blade tissue destined for gene expression analysis was frozen immediately in liquid N_2_ and stored at −80 °C, and the tissue for carbohydrate analysis was lyophilized.

In both years, ears were harvested at the same time as ear-leaf blades and separated into apical and basal halves and cob and ovaries or kernels. These samples were freeze-dried, ground with a coffee grinder and Geno/Grinder 2010 (SPEX SamplePrep), and later used for carbohydrate analysis.

### Gas exchange measurements

In each plot, ear leaves from three to four plants were used to measure net photosynthesis between 09.00 and 12.00 h at silking in both years and 20 DAS in 2015. Measurements were performed near the middle of the leaf blade avoiding the midrib with a portable photosynthesis system (LI-6400; LI-COR, Inc.) and a LI-6400-02B LED light source. Measurement conditions were set at an irradiance of 1800 µmol m^−2^ s^−1^ (PAR), CO_2_ at 400 mmol m^−2^ s^−1^, flow rate at 500 mmol s^−1^, and the leaf temperature maintained at 30 ± 1 °C.

### TEM preparation and observation

Specimen preparation was performed at the Electron Microscopy Core Facility, University of Missouri as described by Chen and Thelen (2010). Images were acquired with a JEOL JEM 1400 transmission electron microscope (JEOL, Peabody, MA, USA) at 80 kV on a Gatan Ultrascan 1000 CCD camera (Gatan, Inc., Pleasanton, CA, USA). Starch granule and chloroplast morphology were analysed using ImageJ software.

### Sugar and starch quantification

Glucose, fructose, and sucrose were extracted from the ground samples using a modified method of Zhao *et al.* (2010). Briefly, a mixture of 30 mg dried tissue was extracted three times with 80% (v/v) ethanol in an 80 °C water bath for 15 min. Extracts were centrifuged and supernatants were combined, filtered through 0.45 μm pore size nylon membranes, and 20 μl were used for glucose, fructose, and sucrose analysis by HPLC (Shimadzu Corp., Kyoto, Japan) with a refractive index detector (Model RID-10A). The mobile phase consisted of 80% (v/v) acetonitrile in water with a flow rate of 1 ml min^−1^. A Luna 5 µm NH_2_ 100 Å, LC Column 250 × 4.6 mm from Phenomenex (Torrance, CA, USA) was used for the analysis which, was performed with the column maintained at 40 °C. Data acquisition was controlled by LabSolutions software (Shimadzu Corp.).

The pellets remaining from the above extractions were used for starch solubilization according to Zhao *et al.* (2010). α-Amylase (Sigma-Aldrich, A3403) and amyloglucosidase (Sigma-Aldrich, A3042) were used to hydrolyse the pellets. The glucose content was assayed as described above by HPLC and used to calculate starch concentration.

### RNA isolation, reverse transcription, and real time PCR (qPCR) analysis

Total RNA was extracted using an RNeasy Plant Mini kit (Qiagen). RNA quantity and quality were assessed using both a standard agarose gel electrophoretic analysis and a Nanodrop ND-1000 spectrophotometer. Afterwards, the isolated RNA sample was used as template for complementary DNA (cDNA) synthesis using oligo (dTs) and SuperScript^TM^ II Reverse Transcriptase (Invitrogen). For qPCR, PowerUp^TM^ SYBR^TM^ Green Master Mix (Applied Biosystems) was used in the reaction mixture according to the manufacturer’s instructions, and reactions were performed in 96-well plates on an ABI 7500 real-time PCR system with universal cycling conditions (95 °C for 2 min, 40 cycles of 95 °C for 15 s, and 60 °C for 1 min). Primers were designed using NCBI Primer-BLAST and Primer Premier 5.0, and are listed in Supplementary Table S1 at *JXB* online. *Ubiquitin* (Zheng *et al.*, 2017) and *β-Tubulin* (Lin *et al.*, 2014) were used as reference genes. Relative expression values were calculated according to the method of Pfaffl (2001), and the diurnal expression values were expressed as fold changes relative to values of normal N supply (N_200_) at time 00.00 h at silking.

### Statistical analyses

Data were subjected to analysis of variance using Proc ANOVA with SAS package 9.1 (SAS Institute, Cary, NC, USA). Nitrogen rate was treated as the fixed effect and replication as the random effect. Two-tail *t* tests were performed for the starch and chloroplast survey data presented in Fig. 3 and Supplementary Fig. S3. The least significant difference was used to determine treatment differences at a *P*<0.05 level of probability.

## Results

### Influence of N application on maize growth and C and N accumulation

In both years, compared with N_200_ and N_300_, low N availability (N_0_) caused reductions of 37–52%, 33–65%, and 55–64% in maize shoot dry matter, yield, and N content, respectively, at maturity (Table 1). Leaf expansion and net photosynthesis at both silking and 20 or 21 DAS were noticeably lower in N_0_ compared with N_200_ and N_300_ plants. Consistent with the shoot N content, N content of the ear leaves of N_0_ plants was lower (31–52%) than in the other two N treatments. Interestingly, similar C contents (g m^−2^) were observed in N_0_ and N_200_ ear leaves, except at 20 DAS in 2015. Consequently, a 1.3- to 1.9-fold greater ear leaf C to N ratio was observed in the N-deficient plants compared with N-sufficient plants (Table 1). Except for shoot dry matter at maturity in 2015, no statistical differences were observed for the above parameters between plants in the N_200_ and N_300_ treatments (Table 1).

**Table 1.** Effects of nitrogen (N) availability on plant dry matter, grain yield, N uptake at maturity and on the leaf area, photosynthesis, carbon (C) and N status in ear leaf at silking and 20 or 21 days after silking (DAS) in 2014 and 2015

Year	Stage	N treatment					
		N_0_	N _ 200 _	N _ 300 _	N _ 0 _	N _ 200 _	N _ 300 _
		**Shoot dry matter (g plant** ^−**1**^)			**Grain yield (g plant** ^−**1**^)		
2014	Maturity	234.4 b	374.3 a	375.6 a	143.4 b	212.6 a	213.8 a
2015	Maturity	148.0 c	306.1 a	251.9 b	61.6 b	174.2 a	153.6 a
		**Shoot N content (g plant** ^−**1**^)			**Ear leaf area at silking (cm** ^**2**^)		
2014	Maturity	1.4 b	4.0 a	4.0 a	741.4 b	833.1 a	823.9 a
2015	Maturity	1.0 b	2.1 a	2.1 a	434.6 b	517.6 a	493.4 a
		**Photosynthesis (μmol CO**_**2**_ **m** ^−**2**^ **s** ^−**1**^)			**Ear leaf C content (g m** ^−**2**^)		
2014	Silking	25.2 b	33.8 a	33.1 a	19.6 b	21.3 ab	21.8 a
	21 DAS	—	—	—	20.0 b	22.1 ab	23.4 a
2015	Silking	28.5 b	44.2 a	47.2 a	24.9 b	26.0 ab	27.7 a
	20 DAS	26.4 b	35.3 a	36.5 a	24.3 b	29.1 a	29.2 a
		**Ear leaf N content (g m** ^−**2**^)			**C to N ratio in ear leaf**		
2014	Silking	0.8 b	1.2 a	1.1 a	23.3 a	17.3 b	18.3 b
	21 DAS	0.5 b	1.1 a	1.1 a	37.6 a	20.2 b	20.9 b
2015	Silking	0.8 b	1.6 a	1.6 a	30.6 a	15.9 b	17.1 b
	20 DAS	0.9 b	1.6 a	1.6 a	28.3 a	18.6 b	17.7 b

Data are means of four replicates. Means with different letters indicate statistical differences between N levels at each sampling date (*P*<0.05).

### Soluble sugars and starch concentrations of ear leaves

**Fig. 1.**
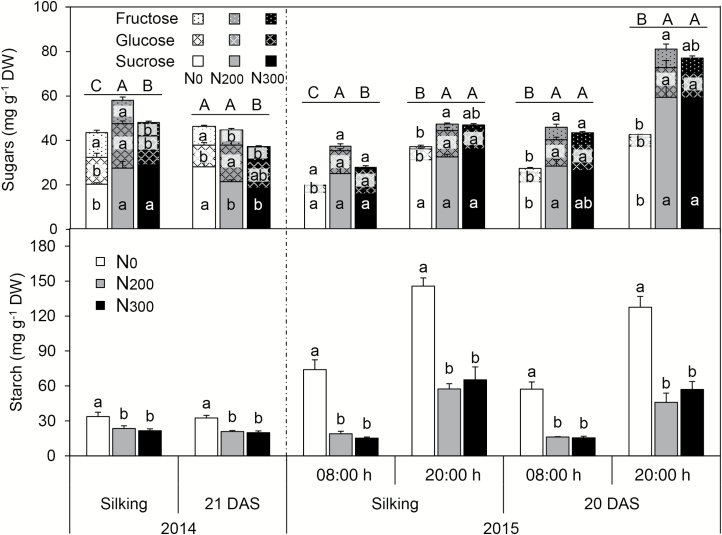
Sugars and starch concentration in ear leaf of maize plants with different nitrogen supplies at silking and 20 or 21 DAS in 2014 and 2015. Ear leaf was collected between 09.00 and 11.00 h in 2014, and at 08.00 and 20.00 h in 2015. Error bars represent the standard error of the mean (*n*=4). Different letters above the columns at each harvest represent the significant differences between nitrogen levels (*P*<0.05).

Nitrogen deficiency significantly reduced the concentration of soluble sugars (sum of fructose, glucose, and sucrose) compared with sufficient N supply, except at 21 DAS in 2014 (Fig. 1). Starch concentration (mg g^−1^) and content (mg leaf^−1^) in ear leaves of N_0_ plants were invariably greater than those from N-sufficient plants, irrespective of the developmental stage and time of day (Fig. 1; Supplementary Fig. S1A). For all the N treatments, ear leaf starch concentrations were greater at 20.00 h than at 08.00 h. In comparison with the N-sufficient plants, N deficiency led to 0.4–0.6-fold increases in mid-morning ear-leaf starch concentration in 2014, and 1.2–1.8-fold increases at 20.00 h and 2.6–3.9-fold increases at 08.00 h in 2015 (Fig. 1). Similar amounts of starch were mobilized during the night in ear leaves of N_0_, N_200_, and N_300_ plants (Supplementary Fig. S1B), corresponding to a degradation percentage of 49%, 67%, and 75%, respectively, at silking, and 48%, 55%, and 69%, respectively, at 20 DAS (Supplementary Fig. S1C). Overall, N deficiency led to an increase of the starch to sucrose ratio in ear leaves of N-deficient plants, although differences were not statistically significant for the assessments conducted mid-morning in 2014 (Supplementary Fig. S2A, B).

### TEM analysis of ear leaf anatomy

**Fig. 2.**
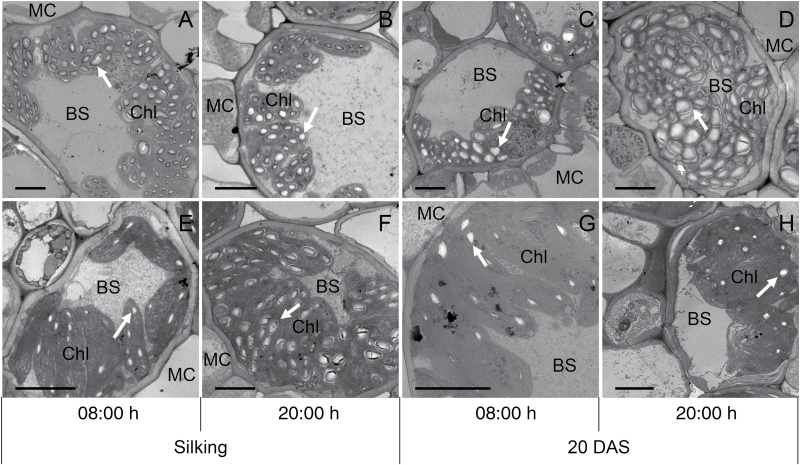
Transmission electron micrographs of bundle sheath cells with starch granules in ear leaves of nitrogen deficient (A–D) and sufficient (E–H) maize plants at 08.00 and 20.00 h of silking and 20 DAS. Arrows indicate the starch granules. BS, bundle sheath cell; Chl, chloroplast; MC, mesophyll cell. Scale bars: 5 μm.

**Fig. 3.**
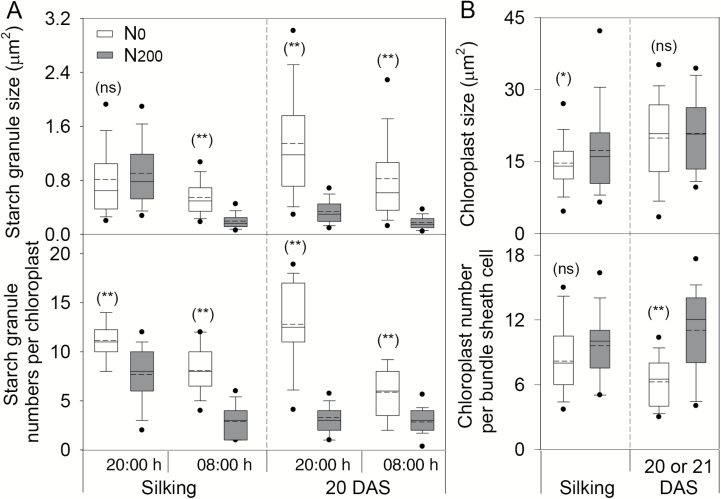
The number and size of starch granules (A), and chloroplasts (B) in bundle sheath cells of maize ear leaf at silking and 20 or 21 DAS in 2014 and 2015. Samples for starch granules observation were collected at 20.00 and 08.00 h in 2015, and the chloroplast data were pooled from 2014 and 2015. The solid line and dashed line within each box represent the median and mean values of all data. The top and bottom edges of the boxes represent the 75 and 25 percentiles, and the top and bottom bars represent the 95 and 5 percentiles of all data, respectively. *P<0.05; **P<0.01; ns, not significant.

Larger starch granules were observed in leaves under N_0_ than N_200_ treatment, except at silking at 20.00 h in 2015 and at silking in 2014 (Figs 2 and 3A; Supplementary Fig. S3A). The starch granule numbers per chloroplast were consistently greater in ear leaves under N deficiency than N sufficiency (Figs 2 and 3A), except at 21 DAS in 2014 when no statistical difference was detected (Supplementary Fig. S3B). Interestingly, smaller diurnal changes of both the size and numbers of starch granules were exhibited in N_200_ leaves at 20 DAS, compared with N_0_ leaves (Fig. 3A). As to the chloroplasts in BS cells, they were smaller in ear leaves of N_0_ than N_200_ plants at silking, but similar in size at 20 or 21 DAS. Fewer chloroplasts were observed in N_0_*vs* N_200_ leaves at 20 or 21 DAS, but not at silking (Fig. 3B).

**Fig. 4.**
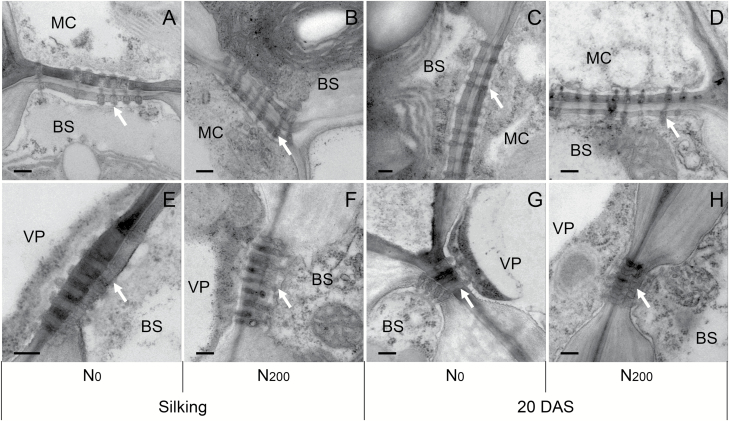
Transmission electron micrographs of plasmodesmata between mesophyll (MC) and bundle sheath (BS) cells, and BS and vascular parenchyma (VP) cells in ear leaves of N_0_ and N_200_ plants at silking and 20 days after silking in 2015. Arrows indicate the plasmodesmata. Scale bars: 200 nm.

Visual examination of plasmodesmata ultrastructure at the interfaces between MC and BS and BS and VP cells was conducted based on TEM images. As shown in Fig. 4, plasmodesmata between MC and BS and BS and VP cells of N_0_ and N_200_ ear leaves collected at silking and 20 DAS in 2015 appeared similar and without occlusions.

### Transcript abundance of genes involving in sucrose and starch metabolism and sucrose export from ear leaves

The MaizeGDB and Gramene databases were used to search for and obtain sequences of genes involved in sucrose and starch metabolism in maize leaves (Supplementary Table S1). The diurnal expression of selected genes was examined using qPCR. *Tpt* (GRMZM2G070605) encodes the triose phosphate/phosphate translocator (TPT) in the inner envelope membrane of the chloroplast that exchanges phosphate and triose phosphate between chloroplast and cytosol. The genes encoding ADP-glucose pyrophosphorylase (AGPase) (*Agpsl1*, *Agpll1*), starch synthase (*Ss1*), β-amylase (*Bmy*), and maltose exporter 1-like (*Mex1-like*) control key steps in starch turnover. Genes encoding sucrose phosphate synthase (*Sps1*), sucrose synthase (*Sus2*), SWEETs (*Sweet13a*, *Sweet13b*, *and Sweet13c*), and SUT1 (*Sut1*) were characterized as they are important for sucrose turnover and export from leaves. For the proteins encoded by multiple genes, the gene with the highest expression in the mature leaf after silking was selected based on the Maize Gene Expression Atlas (Sekhon *et al.* 2011).

**Fig. 5.**
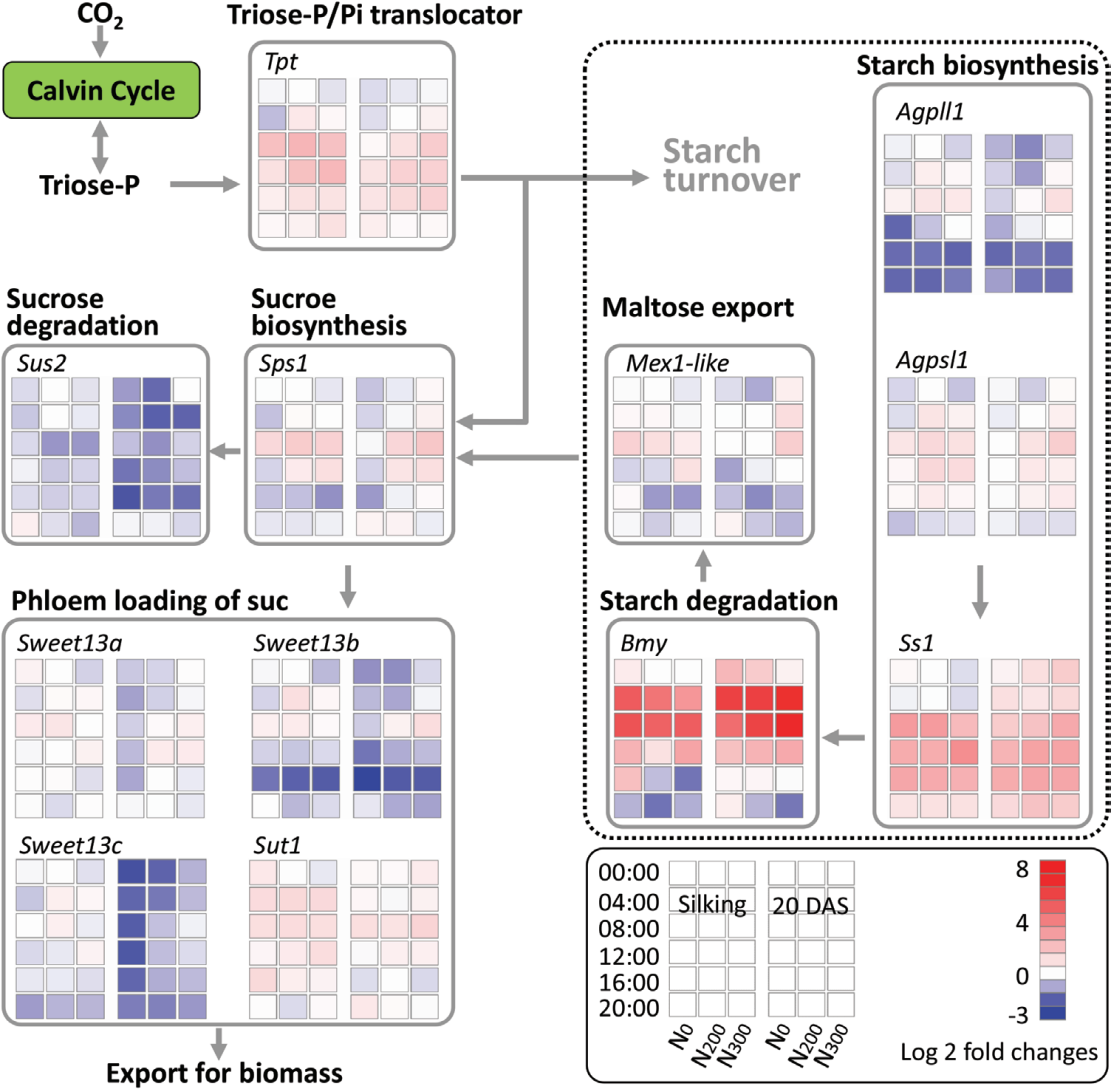
The diurnal changes of relative expression level of genes involved in sucrose and starch turnover or sucrose export from leaves at silking and 20 DAS in 2015. Samples were collected every 4 h during the day and night (*n*=4).

The relative changes in transcript abundance of the different genes in response to N supply and time of day are shown in Fig. 5. Transcript abundance of *Tpt* followed a clear diurnal pattern in all treatments and at both sampling times. In general, *Tpt* expression levels in the N-fertilized treatments were higher and/or increased for an extended period of time compared with the N_0_ treatment. Sucrose phosphate synthase (*Sps1*) expression followed a similar pattern in that expression was usually higher at 08.00 and 12.00 h and generally was lower in N_0_ than in N-fertilized treatments. No distinct expression pattern in the N_0_ compared with the N-fertilized treatments was observed for sucrose synthase (*Sus2*). Of the three SWEETs tested, N fertilization did not appreciably modify the transcript abundance of *Sweet13b*, while *Sweet13a* and *Sweet13c* were down-regulated by N deficiency at 20 DAS, and *Sweet13c* also at silking. *Sut1* expression was high at most time points, and less affected by the N treatment compared with *Sweet13a* and *Sweet13c*.

The *Agpsl1* and *Agpll1* genes encoding enzymes of starch biosynthesis largely were down-regulated, particularly during the photoperiod, in the N_0_ compared with the N_200_ and N_300_ treatments. Although less pronounced, this was also the case for *Ss1* at 20 DAS, but at silking *Ss1* transcript abundance in the N-deficient treatment was very similar to the N-fertilized treatments. β-Amylase transcript levels exhibited a pronounced diurnal pattern at both sampling times, but were not very responsive to N treatment. Its transcript levels were highest at 04.00 and 08.00 h and generally lowest late afternoon (16.00 h) and evening (20.00 h). The expression of maltose exporter 1-like (*Mex1-like*) did not reveal noticeable pattern as related to N treatment, neither at silking nor at 20 DAS.

### Concentrations of sugars and starch in the cob and developing kernels

**Fig. 6.**
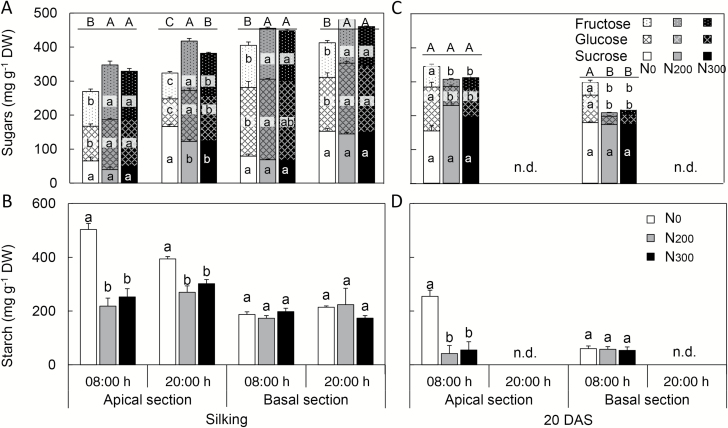
Sugars and starch concentrations in the apical and basal cob sections at silking (A, B) and 20 DAS (C, D) in 2015. Samples were collected at 08.00 and 20.00 h at each harvest. Error bars represent the standard error of the mean (*n*=4). Different upper-case letters above the columns represent the significant differences in total sugars (fructose, glucose and sucrose) between nitrogen treatments, while different lower-case letters indicate the differences for individual sugars or starch between treatments (*P*<0.05). n.d., not determined.

At silking, sucrose levels in the basal cob section were not influenced by N treatment (Fig 6A). In contrast, sucrose levels in the apical cob section were greater in N_0_ than N_200_ and N_300_ treatments at the end of the photoperiod (20.00 h). Due to lower fructose and glucose concentrations in the cob of N-deficient compared with N-sufficient plants, the sum of sugars (sucrose, glucose, and fructose) was lower in both apical and basal cob sections of N_0_ plants than in N_200_ and N_300_ plants (Fig. 6A). Interestingly, starch concentrations in N-deficient plants were greater in the apical cob sections and similar in the basal cob sections to those of N-sufficient plants, both in the morning and in the evening (Fig. 6B).

In contrast to silking, fructose and glucose concentrations at 20 DAS were greater in N_0_ plants than N_200_ and N_300_ plants in both apical and basal cob sections. Unlike fructose and glucose, sucrose concentrations in cobs were similar between N treatments (Fig. 6C). In the basal cob section, the higher fructose and glucose concentrations resulted in greater sugar (sucrose+glucose+fructose) concentrations in N_0_ plants than N-fertilized plants (Fig. 6C). Starch concentrations in the apical cob section in N_0_ treatments were 4-fold greater than in the other two N treatments, but were similar in the basal cob section (Fig. 6D). For all three N treatments, starch concentrations in apical and basal cob sections at 20 DAS were lower than those observed in the respective sections at silking.

**Fig. 7.**
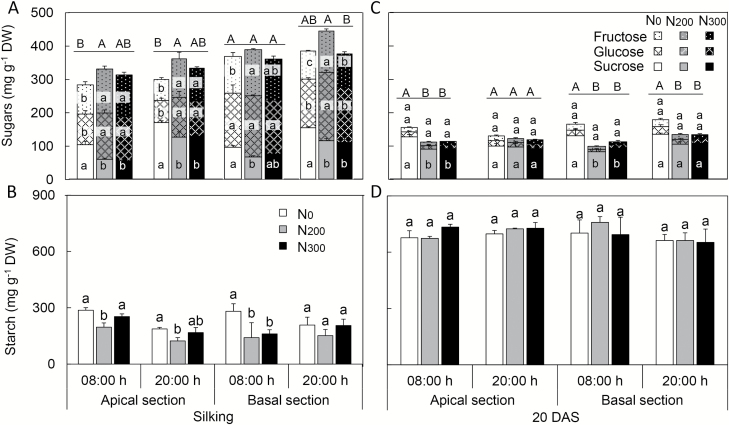
Sugars and starch concentrations in the ovaries (at silking, A, B) and developing kernels (at 20 days after silking, C, D) obtained from apical and basal sections of cobs in 2015. Samples were collected at 08.00 and 20.00 h at each harvest. Error bars represent the standard error of the mean (*n*=4). Different upper-case letters above the columns represent the significant differences in total sugars (fructose, glucose, and sucrose) between nitrogen treatments, while different lower-case letters indicate the differences in each individual sugar or starch between treatments (*P*<0.05).

Not surprisingly, examination of carbohydrate concentrations in developing ovaries (silking) and kernels (20 DAS) revealed dramatic differences in soluble sugar and starch concentrations between these time points (Fig. 7). Compared with N_200_ treatments, N_0_ had lower total soluble sugar concentrations in the apical ovaries but similar in the basal ovaries. Compared with N-sufficient treatments, N_0_ plants exhibited similar or greater starch levels in apical as well as basal ovaries (Fig. 7A, B). Glucose and fructose comprised more than 60% (except N_0_ at 20.00 h) of the soluble sugars in the ovaries at silking, while sucrose was the dominant (>75% of total) soluble sugar in developing kernels from both apical and basal sections. At 20 DAS, except for apical kernels at 20.00 h, N_0_ plants had significantly greater soluble sugar concentrations in both apical and basal kernels than N_200_ and N_300_ plants (Fig. 7C). In contrast, starch concentrations in developing kernels did not differ among N treatments (Fig. 7D).

### Yield components

**Fig. 8.**
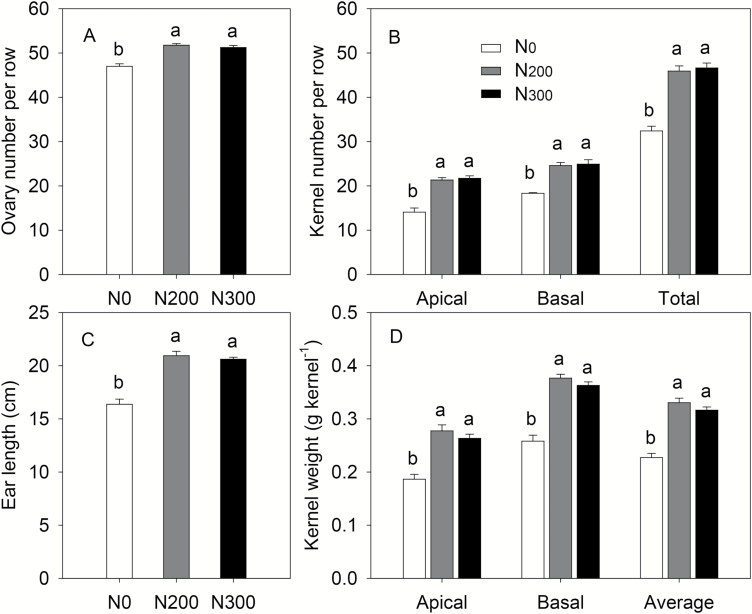
The ovary number at silking (A), apical and basal kernel number per row (B), ear length (C), and apical and basal kernel weight (D) at maturity in maize plants supplied with different N levels in 2014. Error bars represent the standard error of the mean (*n*=4). Different letters above the columns represent significant differences between nitrogen levels (*P*<0.05).

The number of ovaries per row was significantly greater in N_200_ and N_300_ treatments than in the N_0_ treatment. These differences in ovary numbers at silking also translated into a lower number of kernels per row at maturity in the N_0_ treatment (Fig. 8A, B). In N_0_ plants, 31% of ovaries were aborted after silking, while 9–11% were aborted in N_200_ and N_300_ plants (Fig. 8A, B). Ears from N-sufficient plants were 26–28% longer than those from N_0_ plants (Fig. 8C). As compared with N_0_, applications of 200 or 300 kg N ha^−1^ increased kernel weights by 39–49%, with a similar effect for apical and basal kernels (Fig. 8D).

## Discussion

### Greater starch accumulation in N-deficient leaves is associated with more and larger starch granules in bundle sheath cells

Consistent with previous reports (Peng *et al.*, 2013, 2014), N deficiency significantly stunted maize growth and led to greater starch accumulation in leaves (Table 1; Fig. 1; Supplementary Fig. S1). As a storage carbohydrate, starch does not demonstrate regulatory activities, but is a major integrator in the regulation of plant growth (Sulpice *et al.*, 2009). For example, starch synthesis competes with sucrose synthesis for triose-P_i_, a shared substrate for the two biochemical reactions (Stitt and Zeeman, 2012). Triose-P_i_ is transported from the stroma to the cytoplasm by triose-P_i_/P_i_ translocators (TPT) localized on the inner envelope membrane of the chloroplast, and the activity of TPT proteins is a key factor influencing carbon flux between sucrose and starch (Stitt and Zeeman, 2012). In leaves of N-deficient plants, the transcript abundance of *Tpt* was lower than in N-sufficient (N_200_ and N_300_) plants for most of the daylight hours at both silking and 20 DAS (Fig. 5), suggesting lower TPT abundance and triose-P_i_ transport in the N-deficient plants. Limited transport of triose-P_i_ into the cytosol is consistent with greater C flow to starch synthesis in the chloroplast at the cost of cytosolic synthesis of sucrose. This is in accord with the greater starch to sucrose ratio observed in leaves of N-deficient than N-sufficient plants (Supplementary Fig. S2).

Examination of transmission electron micrographs of samples collected 20 DAS revealed fewer but similar sized chloroplasts in the bundle sheath of ear leaves from N_0_ compared with N_200_ plants (Figs 2 and 3). In addition, starch granules were only found in the BS cells of mature leaves, and their size and number per chloroplast were greater in leaves of N-deficient compared with N-sufficient plants, thus at least partially explaining the greater starch concentrations and contents of N_0_ ear leaves. Assessments of starch granule number and size in ear leaves sampled at 08.00 and 20.00 h revealed larger and more numerous starch granules per chloroplast in the evening *vs* morning for N_0_ leaves at both silking and 20 DAS. While a dramatic diurnal difference was also observed in N_200_ ear leaves at silking, differences were limited for starch granule size and absent for granule number per chloroplast at 20 DAS. These results indicate that the diurnal pattern of starch granule size and number in maize is N responsive and stage dependent, which is in contrast to the report by Crumpton-Taylor *et al.* (2012) that the diurnal changes of starch levels in Arabidopsis are largely influenced by changes in the granule size with fixed number of granules.

Previously, Zhao *et al.* (2015) reported that both gene expression levels and enzyme activities in N-deficient duckweed plants are consistent with enhanced starch biosynthesis and inhibited starch degradation. In the present study, the transcript levels of enzymes involved in starch biosynthesis and degradation were either lower in N-deficient than N-sufficient ear leaves, or largely unaltered by N treatment. These results are consistent with other reports that starch metabolism-related transcripts are repressed under N starvation in maize (Schlüter *et al.*, 2012), indicating reduced starch turnover in the N-deficient plants. Additionally, the lower transcript levels of starch synthesis genes is consistent with the significantly lower photosynthesis in N-starved leaves, i.e. a reduced total C flux under N deficiency (Radin and Eidenbock, 1986). Among all the genes examined in this study, the most pronounced diurnal changes in transcript levels were observed for β-amylase, which is involved in starch degradation. Significant up-regulation of β-amylase expression was found at 04.00 h and 08.00 h in all N treatments. However, while strong diurnal regulation was observed, β-amylase expression in ear leaves was largely unresponsive to N treatment, which was consistent with the observation that several enzymes involved in starch turnover are under post-translational or redox regulation (Hendriks *et al.*, 2003; Mikkelsen *et al.*, 2005; Sparla *et al.*, 2006). Similar to β-amylase expression, no substantial effect of N treatment on *Mex1-like* transcript abundance was observed, suggesting limited if any influence of N treatment on maltose export from chloroplasts. This absence of a strong N effect on *Mex1-like* expression is aligned with similar amounts of starch mobilization from N-deficient and N-sufficient ear leaves. However, since the percentage of starch degraded in N-deficient leaves tended to be lower than in N-sufficient leaves, and larger and more residual starch granules remained at the end of the night (Figs 2 and 3; Supplementary Fig. S1), we speculate that leaf starch mobilization was perturbed by N deficiency.

Transitory starch accumulation acts as an overflow mechanism during the day when assimilation exceeds sucrose synthesis, and is mobilized for respiration and growth at night (Fig. 1; Stitt and Zeeman, 2012). Mutations blocking either starch synthesis or starch breakdown in Arabidopsis reduce plant growth (Stitt and Zeeman, 2012). In maize, a leaf starchless mutant, *agps-m1*, has diminished growth and productivity under field conditions (Schlosser *et al.*, 2012). Greater transient starch accumulation has been observed in response to not only low N availability but also other abiotic stress conditions that reduce carbon demand, including low temperature, low light intensity and low P supply (Schlüter *et al.*, 2012; Zhao *et al.*, 2015), suggesting a pivotal role of sink utilization in the regulation of starch turnover. The sugar signaling molecule trehalose 6-phosphate and SnRK1 protein kinase may mediate the feedback regulation of starch synthesis and degradation via modulation of the sucrose status in leaves (Martins *et al.*, 2013).

### The symplastic pathway of sucrose movement in N-deficient leaves is open and expression levels of sucrose transporters tended to be lower than in N-sufficient leaves

Yield, shoot dry matter, leaf area, and photosynthesis data revealed a pronounced N limitation for plants in the N_0_ compared with N_200_ and N_300_ treatments (Table 1). Except for 21 DAS in 2014, N limitation as measured by these indicators was associated with lower soluble sugar concentrations in ear leaves (Fig. 1). For the most part, the low soluble sugar concentrations were a function of lower sucrose and glucose concentrations in ear leaves of N_0_ compared with N-sufficient plants. As discussed above, N availability has noticeable effects on C partitioning between starch and sucrose in leaves. Peng *et al.* (2013, 2014) observed greater starch concentrations in source leaves under N deficiency and suggested that carbohydrate availability *per se* may not be the yield-limiting factor but that C mobilization and transport out of the leaves may constrain kernel development and yield under N deficiency. However, it remains to be shown whether C mobilization and export from source leaves limit yield of maize under N-deficient conditions. In maize, sucrose is synthesized in the cytosol of leaf cells and then moves symplastically from MC to BS to VP cells through plasmodesmata (Braun and Slewinski, 2009). In the maize *sxd1* mutant, sucrose movement is blocked by callose deposition at the BS side of BS–VP interfaces, and starch and soluble sugars accumulate in leaves (Russin *et al.* 1996; Ma *et al.*, 2008). Interestingly, Kong *et al.* (2013) reported that N deficiency compared with normal N supply leads to greater deposition of callose in wheat peduncle vascular bundles during mid grain filling, and that this partly contributes to reduced yields. However, scrutiny of the transmission electron micrographs obtained in the present study did not reveal physical blockage of plasmodesmata at the interfaces of MC, BS and VP cells in N-deficient ear leaves. N_0_ and N_200_ plants had conspicuous and visually normal plasmodesmata appearances at both silking and 20 DAS (Fig. 4), implying that the symplastic pathway of sucrose transport between different types of cells in leaves is open in N-deficient plants.

Maize is an apoplastic phloem loading species in which, following symplastic transport to the VP cells, sucrose is moved into the apoplast by sugar effluxers, SWEETs (Chen *et al.*, 2012), and loaded into the phloem by SUTs (Slewinski *et al.*, 2009; Baker *et al.*, 2016). Interestingly, of the three *Sweet* genes examined in this study, transcript levels of *Sweet13a* and *Sweet13c* were lower during the day in N_0_ compared with N_200_ and N_300_ leaves, especially during day-time hours at 20 DAS (Fig. 5). Reduced *Sweet13* transcript levels in the N_0_ treatment may indicate fewer SWEET proteins and lower sucrose efflux to the intercellular space. As for the expression of *ZmSut1*, the transcript levels did not follow the pattern observed for the SWEET transporters in that no consistent N treatment effects were found. The lack of responsiveness to N supply may suggest post-translational regulation of SUT1 in the examined tissues. In fact, such regulation by redox conditions has been documented in potato (*Solanum tuberosum*) (Krügel *et al.*, 2008).

### Higher carbohydrate concentrations in the cob or developing kernels imply that the long distance transport of sugars is not a main limiting factor for yield under N deficiency

To elucidate carbohydrate dynamics in the cob and ovaries or kernels, soluble sugar and starch concentrations were examined in these tissues at silking and 20 DAS. Sugar, in maize largely sucrose, is transported via the phloem to the cob where photoassimilates and other nutrients are temporarily stored. From there, sucrose is largely cleaved by vacuolar or cell wall invertases into fructose and glucose in the pedicel, the placenta–chalaza, and especially by cell wall invertases in the basal endosperm transfer layer (Shannon, 1972; Bihmidine *et al.*, 2013). At the early reproductive stage, sucrose cleavage by invertases is a key factor for sink strength establishment, since invertases participate not only in the sucrose metabolism, but also in the production of the hexose signals that regulate cell cycle and cell division programs (reviewed by Bihmidine *et al.*, 2013).

Compared with N-sufficient plants, similar or higher sucrose concentration but lower fructose and glucose concentrations were observed in the apical cob section of N-deficient plants (Fig. 6), suggesting that sucrose cleavage may be altered in this maternal tissue. The considerably greater starch accumulation in the apical cob section of N-deficient plants suggests that more sucrose is used for synthesis of transient starch instead of translocation into ovaries in these plants. Consistent with this observation, fructose and glucose levels in the apical ovaries were lower in N-deficient compared with N-sufficient plants at silking (Fig. 7). Meanwhile, greater starch accumulation in the apical cob section at silking implies that supply of sugars from source leaves may not be the primary limitation under N deficiency, and points to a limitation associated with the movement of cleaved sugars into ovaries. These observations suggest that the amount of sugars arriving in the ear under N-deficient conditions exceeds the capacity of the sinks to utilize them. Previous research also showed that N deficiency reduces the number of cells and starch granules in the endosperm, and represses the activity of enzymes regulating sucrose uptake or utilization, such as soluble and bound invertase, sucrose synthase, and AGPase (Singletary and Below, 1990; Singletary *et al.*, 1990; Cazetta *et al.*, 1999). Recent molecular analysis revealed that ZmSWEET4c functions in hexose translocation in the basal endosperm transfer layer, and further examination and characterization of ZmSWEET4c can be expected to shed light on hexose transport into the developing kernels, including in relation to dynamics in N-deficient compared with N-sufficient plants (Sosso *et al.*, 2015).

### Limited ability to utilize sugars in ears under N deficiency in turn represses photosynthesis and sucrose export from source leaves

Improvement of crop yields involves enhanced allocation of assimilates from source to sink, which is regulated by a complex signaling network encompassing sugars, phytohormones, environmental factors, etc. (Yu *et al.*, 2015). In the absence of fertilizer application, limited N can stifle photosynthesis in source leaves and/or limit the utilization of assimilates in sink organs and thus constrain yield. As N is an essential component of proteins and chlorophyll, limited N supply constrains photosynthesis and thus the amount of fixed C available for growth and yield formation (Radin and Eidenbock, 1986). In addition, the suppression of leaf chlorophyll content, photosynthesis and sucrose production by N deficiency before silking has been well documented (Lu and Zhang, 2000). Reduced sucrose production in the leaf could lead to reduced ovary initiation, lower ear growth rate, and increased ovary abortion (Jacobs and Pearson, 1992; Andrade *et al.*, 2002), as well as delayed flowering, slow silk emergence, and increased anthesis–silking interval (Hanway, 1962; Lafitte and Edmeades, 1994; Lemcoff and Loomis, 1994), which further limits sink strength during grain filling, and imposes a sink to source feedback regulation on leaf carbon accumulation and export.

It is clear that photosynthesis is under feedback control, and that the C to N balance is more important than C status *per se* to understand C metabolite feedback control of photosynthesis (Paul and Pellny, 2003). Under N deficiency, amino acid assimilation is suppressed, thus leading to reduced demand for C skeletons (amino acid precursors such as malate, citrate, and 2-oxoglutarate) (Scheible *et al.*, 1997; Paul and Pellny, 2003), followed by greater C flux into transitory starch for storage rather than into sucrose synthesis (Supplementary Fig. S2). This may be mediated by feedback inhibition of sucrose synthesis via the signal metabolite fructose-2,6-bisphosphate that leads to the accumulation of phosphorylated intermediates and decreased inorganic phosphate in the chloroplast, resulting in allosteric activation of AGPase by a rising glycerate-3-phosphate to P_i_ ratio, in turn stimulating starch biosynthesis (Stitt and Zeeman, 2012). Along with lower sucrose accumulation in leaves, sucrose exporter expression (primarily *Sweet13a* and *Sweet13c*) in N-deficient leaves was down-regulated for much of the photoperiod in the present study, suggesting reduced sucrose export from leaves likely due to diminished sink C demand. As well, while leaf soluble sugar levels measured at 08.00 and 20.00 h in N-deficient conditions were lower than in N-sufficient treatments at 20 DAS in 2015 (Fig. 1), this was not the case when measured late morning at 21 DAS in 2014, which is in accordance with diurnal dynamics associated with diminished sink demand in the late morning in N-deficient plants. The observed diurnal and N treatment-related changes in sucrose transporter expression are consistent with dynamic regulation of sugar transport in apoplastic phloem loading species (Slewinski and Braun, 2010).

## Conclusions

Reduced photosynthesis in ear leaves of N-deficient as compared with N-sufficient plants was associated with more C flow into transitory starch and reduced sugar export out of leaves. The greater starch concentrations in N-deficient leaves were associated with larger and more numerous starch granules in bundle sheath chloroplasts. Higher starch concentrations in N-deficient apical cob tissue at silking and 20 DAS suggested that photoassimilate availability from source leaves was not the main factor limiting kernel growth. Generally, higher soluble sugar concentrations in the developing kernels (20 DAS) of N-deficient plants indicated limitations in the utilization of available sugars. In combination with the high soluble sugar or starch concentrations in the cob at 20 DAS, these results also indicated that reduced utilization in the developing kernels may limit transport from the cob to the kernels, which in turn may impose a feedback inhibition on photosynthesis and sugar export from leaves. However, since no physical blockage of plasmodesmata was observed, N deficiency did not appear to affect the symplastic pathway. Further research is needed to elucidate the mechanisms underlying carbohydrate accumulation in leaf, cob, and developing kernel tissues, in particular in relation to hexose translocation in the basal endosperm transfer layer and the utilization of soluble sugars with regard to kernel filling and ultimately yield under N-deficient conditions.

## Supplementary data

Supplementary data are available at *JXB* online.

Fig. S1. The amounts of starch accumulation and degradation in ear leaves of maize under different nitrogen supply.

Fig. S2. Starch to sucrose ratio in maize ear leaves at silking and 20 or 21 d after silking.

Fig. S3. The size (A) and number (B) of starch granules in bundle sheath cells of maize ear leaf at 21 d after silking in 2014.

Table S1. List of the primers used in this research.

Supplementary Table Figures

## Abbreviations

BS: bundle sheath
C: carbon
DAS: days after silking
MC: mesophyll cells
N: nitrogen
P_i_: inorganic phosphate
VP: vascular parenchyma.

## References

[CIT0001] Andrade FH, Echarte L, Rizzalli R, Della Maggiora A, Casanovas M 2002 Kernel number prediction in maize under nitrogen or water stress. Crop Science 42, 1173–1179.

[CIT0002] Baker RF, Leach KA, Boyer NR, Swyers MJ, Benitez-Alfonso Y, Skopelitis T, Luo A, Sylvester A, Jackson D, Braun DM 2016 Sucrose transporter *ZmSut1* expression and localization uncover new insights into sucrose phloem loading. Plant Physiology 172, 1876–1898. 27621426 10.1104/pp.16.00884 PMC5100798

[CIT0003] Bihmidine S, Hunter CT 3rd, Johns CE, Koch KE, Braun DM 2013 Regulation of assimilate import into sink organs: update on molecular drivers of sink strength. Frontiers in Plant Science 4, 177. 23761804 10.3389/fpls.2013.00177 PMC3671192

[CIT0004] Braun DM, Slewinski TL 2009 Genetic control of carbon partitioning in grasses: roles of sucrose transporters and tie-dyed loci in phloem loading. Plant Physiology 149, 71–81. 19126697 10.1104/pp.108.129049 PMC2613709

[CIT0005] **CARES** 2012 The Cooperative Soil Survey Columbia, MO: Center for Applied Research and Engagement Systems (CARES) https://cares.missouri.edu/.

[CIT0006] Cazetta JO, Seebauer JR, Below FE 1999 Sucrose and nitrogen supplies regulate growth of maize kernels. Annals of Botany 84, 747–754.

[CIT0007] Chen LQ, Qu XQ, Hou BH, Sosso D, Osorio S, Fernie AR, Frommer WB 2012 Sucrose efflux mediated by SWEET proteins as a key step for phloem transport. Science 335, 207–211. 22157085 10.1126/science.1213351

[CIT0008] Chen M, Thelen JJ 2010 The plastid isoform of triose phosphate isomerase is required for the postgerminative transition from heterotrophic to autotrophic growth in *Arabidopsis*. The Plant Cell 22, 77–90. 20097871 10.1105/tpc.109.071837 PMC2828694

[CIT0009] Crumpton-Taylor M, Grandison S, Png KM, Bushby AJ, Smith AM 2012 Control of starch granule numbers in Arabidopsis chloroplasts. Plant Physiology 158, 905–916. 22135430 10.1104/pp.111.186957 PMC3271777

[CIT0010] Hawkesford M, Horst W, Kichey T, Lambers H, Schjoerring J, Skrumsager Møller I, White P 2012 Functions of macronutrients. In: Marschner P, ed. Marschner’s mineral nutrition of higher plants. 3rd edn, Amsterdam: Elsevier, 135–151.

[CIT0011] Hanway JJ 1962 Com growth and composition in relation to soil fertility. I. Growth of different plant parts and relation between leaf weight and grain yield. Agronomy Journal 54, 145–148.

[CIT0012] Hendriks JH, Kolbe A, Gibon Y, Stitt M, Geigenberger P 2003 ADP-glucose pyrophosphorylase is activated by posttranslational redox-modification in response to light and to sugars in leaves of Arabidopsis and other plant species. Plant Physiology 133, 838–849. 12972664 10.1104/pp.103.024513 PMC219057

[CIT0013] Jacobs BC, Pearson CJ 1992 Pre-flowering growth and development of the inflorescences of maize I. Primordia production and apical dome volume. Journal of Experimental Botany 43, 557–563.

[CIT0014] Jeannette E, Rocher JP, Prioul JL 1995 Effect of an increased sink demand on the carbon metabolism and export of a maize source leaf. Physiologia Plantarum 94, 319–327.

[CIT0015] Kong L, Wang F, Si J, Feng B, Zhang B, Li S, Wang Z 2013 Increasing in ROS levels and callose deposition in peduncle vascular bundles of wheat (*Triticum aestivum* L.) grown under nitrogen deficiency. Journal of Plant Interactions 8, 109–116.

[CIT0016] Krügel U, Veenhoff LM, Langbein J, Wiederhold E, Liesche J, Friedrich T, Grimm B, Martinoia E, Poolman B, Kühn C 2008 Transport and sorting of the *Solanum tuberosum* sucrose transporter SUT1 is affected by posttranslational modification. The Plant Cell 20, 2497–2513. 18790827 10.1105/tpc.108.058271 PMC2570718

[CIT0017] Lafitte HR, Edmeades GO 1994 Improvement for tolerance to low nitrogen in tropical maize. I. Selection criteria. Field Crops Research 39, 1–14.

[CIT0018] Lee EA, Tollenaar M 2007 Physiological basis of successful breeding strategies for maize grain yield. Crop Science 47, 202–215.

[CIT0019] Lemcoff JH, Loomis RS 1994 Nitrogen and density influences on silk emergence, endosperm development, and grain yield in maize (*Zea mays* L.). Field Crops Research 38, 63–72.

[CIT0020] Lin Y, Zhang C, Lan H, Gao S, Liu H, Liu J, Cao M, Pan G, Rong T, Zhang S 2014 Validation of potential reference genes for qPCR in maize across abiotic stresses, hormone treatments, and tissue types. PLoS ONE 9, e95445. 24810581 10.1371/journal.pone.0095445 PMC4014480

[CIT0021] Lu C, Zhang J 2000 Photosynthetic CO_2_ assimilation, chlorophyll fluorescence and photoinhibition as affected by nitrogen deficiency in maize plants. Plant Science 151, 135–143. 10808069 10.1016/s0168-9452(99)00207-1

[CIT0022] Ma Y, Baker RF, Magallanes-Lundback M, DellaPenna D, Braun DM 2008 *Tie-dyed1* and *sucrose export defective1* act independently to promote carbohydrate export from maize leaves. Planta 227, 527–538. 17924136 10.1007/s00425-007-0636-6 PMC2249615

[CIT0023] Mäkelä P, McLaughlin JE, Boyer JS 2005 Imaging and quantifying carbohydrate transport to the developing ovaries of maize. Annals of Botany 96, 939–949. 16100223 10.1093/aob/mci246 PMC4247060

[CIT0024] Martins MC, Hejazi M, Fettke J et al 2013 Feedback inhibition of starch degradation in Arabidopsis leaves mediated by trehalose 6-phosphate. Plant Physiology 163, 1142–1163. 24043444 10.1104/pp.113.226787 PMC3813640

[CIT0025] Masclaux-Daubresse C, Daniel-Vedele F, Dechorgnat J, Chardon F, Gaufichon L, Suzuki A 2010 Nitrogen uptake, assimilation and remobilization in plants: challenges for sustainable and productive agriculture. Annals of Botany 105, 1141–1157. 20299346 10.1093/aob/mcq028 PMC2887065

[CIT0026] Mikkelsen R, Mutenda KE, Mant A, Schurmann P, Blennow A 2005 α-Glucan, water diknase (GWD): A plastidic enzyme with redox regulated and coordinated catalytic activity and binding affinity. Proceedings of the National Academy of Sciences, USA 102, 1785–1790. 10.1073/pnas.0406674102 PMC547843 15665090

[CIT0027] Ning P, Li S, Yu P, Zhang Z, Li CJ 2013 Post-silking accumulation and partitioning of dry matter, nitrogen, phosphorus and potassium in maize varieties differing in leaf longevity. Field Crops Research 144, 19–27.

[CIT0028] Paul MJ, Pellny TK 2003 Carbon metabolite feedback regulation of leaf photosynthesis and development. Journal of Experimental Botany 54, 539–547. 12508065 10.1093/jxb/erg052

[CIT0029] Peng Y, Li C, Fritschi FB 2013 Apoplastic infusion of sucrose into stem internodes during female flowering does not increase grain yield in maize plants grown under nitrogen-limiting conditions. Physiologia Plantarum 148, 470–480. 23061679 10.1111/j.1399-3054.2012.01711.x

[CIT0030] Peng Y, Li C, Fritschi FB 2014 Diurnal dynamics of maize leaf photosynthesis and carbohydrate concentrations in response to differential N availability. Environmental and Experimental Botany 99, 18–27.

[CIT0031] Peng Y, Zeng X, Houx JH, Boardman DL, Li C, Fritschi FB 2016 Pre- and post-silking carbohydrate concentrations in maize ear-leaves and developing ears in response to nitrogen availability. Crop Science 56, 3218–3227.

[CIT0032] Pfaffl MW 2001 A new mathematical model for relative quantification in real-time RT-PCR. Nucleic Acids Research 29, e45. 11328886 10.1093/nar/29.9.e45 PMC55695

[CIT0033] Pilkington SM, Encke B, Krohn N, Höhne M, Stitt M, Pyl ET 2015 Relationship between starch degradation and carbon demand for maintenance and growth in *Arabidopsis thaliana* in different irradiance and temperature regimes. Plant, Cell & Environment 38, 157–171. 10.1111/pce.12381 24905937

[CIT0034] Radin JW, Eidenbock MP 1986 Carbon accumulation during photosynthesis in leaves of nitrogen- and phosphorus-stressed cotton. Plant Physiology 82, 869–871. 16665124 10.1104/pp.82.3.869 PMC1056222

[CIT0035] Russin WA, Evert RF, Vanderveer PJ, Sharkey TD, Briggs SP 1996 Modification of a specific class of plasmodesmata and loss of sucrose export ability in the *sucrose export defective1* maize mutant. The Plant Cell 8, 645–658. 12239395 10.1105/tpc.8.4.645 PMC161126

[CIT0036] Scheible WR, Gonzalez-Fontes A, Lauerer M, Muller-Rober B, Caboche M, Stitt M 1997 Nitrate acts as a signal to induce organic acid metabolism and repress starch metabolism in tobacco. The Plant Cell 9, 783–798. 12237366 10.1105/tpc.9.5.783 PMC156956

[CIT0037] Schlosser AJ, Martin JM, Hannah LC, Giroux MJ 2012 The maize leaf starch mutation *agps-m1* has diminished field growth and productivity. Crop Science 52, 700–706.

[CIT0038] Schlüter U, Mascher M, Colmsee C, Scholz U, Bräutigam A, Fahnenstich H, Sonnewald U 2012 Maize source leaf adaptation to nitrogen deficiency affects not only nitrogen and carbon metabolism but also control of phosphate homeostasis. Plant Physiology 160, 1384–1406. 22972706 10.1104/pp.112.204420 PMC3490595

[CIT0039] Sekhon RS, Lin H, Childs KL, Hansey CN, Buell CR, de Leon N, Kaeppler SM 2011 Genome-wide atlas of transcription during maize development. The Plant Journal 66, 553–563. 21299659 10.1111/j.1365-313X.2011.04527.x

[CIT0040] Shannon JC 1972 Movement of ^14^C-labeled assimilates into kernels of *Zea mays* L: I. Pattern and rate of sugar movement. Plant Physiology 49, 198–202. 16657924 10.1104/pp.49.2.198 PMC365928

[CIT0041] Singletary GW, Below FE 1990 Nitrogen-induced changes in the growth and metabolism of developing maize kernels grown in vitro. Plant Physiology 92, 160–167. 16667240 10.1104/pp.92.1.160 PMC1062264

[CIT0042] Singletary GW, Doehlert DC, Wilson CM, Muhitch MJ, Below FE 1990 Response of enzymes and storage proteins of maize endosperm to nitrogen supply. Plant Physiology 94, 858–864. 16667863 10.1104/pp.94.3.858 PMC1077313

[CIT0043] Slewinski TL, Braun DM 2010 Current perspectives on the regulation of whole-plant carbohydrate partitioning. Plant Science 178, 341–349.

[CIT0044] Slewinski TL, Meeley R, Braun DM 2009 *Sucrose transporter1* functions in phloem loading in maize leaves. Journal of Experimental Botany 60, 881–892. 19181865 10.1093/jxb/ern335 PMC2652052

[CIT0045] Sosso D, Luo D, Li QB et al 2015 Seed filling in domesticated maize and rice depends on SWEET-mediated hexose transport. Nature Genetics 47, 1489–1493. 26523777 10.1038/ng.3422

[CIT0046] Sparla F, Costa A, Lo Schiavo F, Pupillo P, Trost P 2006 Redox regulation of a novel plastid-targeted β-amylase of Arabidopsis. Plant Physiology 141, 840–850. 16698902 10.1104/pp.106.079186 PMC1489908

[CIT0047] Stitt M, Zeeman SC 2012 Starch turnover: pathways, regulation and role in growth. Current Opinion in Plant Biology 15, 282–292. 22541711 10.1016/j.pbi.2012.03.016

[CIT0048] Sulpice R, Pyl ET, Ishihara H et al 2009 Starch as a major integrator in the regulation of plant growth. Proceedings of the National Academy of Sciences, USA 106, 10348–10353. 10.1073/pnas.0903478106 PMC2693182 19506259

[CIT0049] Wang L, Lu Q, Wen X, Lu C 2015 Enhanced sucrose loading improves rice yield by increasing grain size. Plant Physiology 169, 2848–2862. 26504138 10.1104/pp.15.01170 PMC4677907

[CIT0050] Wang R, Tischner R, Gutiérrez RA, Hoffman M, Xing X, Chen M, Coruzzi G, Crawford NM 2004 Genomic analysis of the nitrate response using a nitrate reductase-null mutant of Arabidopsis. Plant Physiology 136, 2512–2522. 15333754 10.1104/pp.104.044610 PMC523318

[CIT0051] Yu SM, Lo SF, Ho TH 2015 Source-sink communication: Regulated by hormone, nutrient, and stress cross-signaling. Trends in Plant Science 20, 844–857. 26603980 10.1016/j.tplants.2015.10.009

[CIT0052] Zhao D, MacKown CT, Starks PJ, Kindiger BK 2010 Rapid analysis of nonstructural carbohydrate components in grass forage using microplate enzymatic assays. Crop Science 50, 1537–1545.

[CIT0053] Zhao Z, Shi HJ, Wang ML, Cui L, Zhao H, Zhao Y 2015 Effect of nitrogen and phosphorus deficiency on transcriptional regulation of genes encoding key enzymes of starch metabolism in duckweed (*Landoltia punctata*). Plant Physiology and Biochemistry 86, 72–81. 25438139 10.1016/j.plaphy.2014.11.007

[CIT0054] Zheng H, Wu H, Pan X, Jin W, Li X 2017 Aberrant meiotic modulation partially contributes to the lower germination rate of pollen grains in maize (*Zea mays* L.) under low nitrogen supply. Plant & Cell Physiology 58, 342–353. 28007967 10.1093/pcp/pcw195

