# Supplementary material for: Post-silking carbon partitioning under nitrogen deficiency revealed sink limitation of grain yield in maize
Source: J Exp Bot. 2018 Jan 18;69(7):1707–19. doi: 10.1093/jxb/erx496 (PMC5888971; doi:10.1093/jxb/erx496)
Supplement: Supplementary Table Figures [file erx496_suppl_table_s1_figures_s1-s3.pdf]

Supplementary data

Table S1. List of primers used in this research.

Gene	Primer sequences (5'-3')
Tpt (GRMZM2G070605)	Fw: GGAGAAAAGGAAAAAGAGCGCAT
	Re: ACGATGTTGTTGTTGTGTCCC
Agpsl1 (GRMZM2G163437)	Fw: TGGGACAGTTATATGAAGCGGA
	Re: TCCATGATCTCCAGCACACTG
Agpll1 (GRMZM2G391936)	Fw: TCCTAAACCTTCTAAGATGGCG
	Re: AAACTGAACCTTGGAGGCTGT
Ss1 (GRMZM2G129451)	Fw: GCTATTGGCTCCATTGCTCC
	Re: TTCAAAACTGTCGTTCGGCT
Bmy (GRMZM2G082034)	Fw: CCCATGAGGACGACCTGCCA
	Re: TTTATCACCCGCCCGTTTATTTTG
Mex1-like (GRMZM2G156356)	Fw: GGTTGGACAGCCACACTTCT
	Re: TGCAGAACCAGTGAACCACA
Sps1 (GRMZM5G875238)	Fw: CGTTCCTCATCAAAGACCCCC
	Re: ACGGAAAGATACCTGAGTGCCT
Sus2 (GRMZM2G318780)	Fw: CCAACCGCAGTAGTAATGGC
	Re: CGGCTTGCCAGCAAAGAAAT
Sweet13a (GRMZM2G173669)	Fw: CTGGGCGTTTGCTTTCG
	Re: ACTTGCTCTTGTAGATGCGGTA
Sweet13b (GRMZM2G021706)	Fw: TGCGTACTGCGTAGTTCCAT
	Re: GGAGATGACGTTGCCTAGGAG
Sweet13c (GRMZM2G179349)	Fw: CAAGAGTTTGAGACAGCAGAGG
	Re: CCAGGAAGGTCATGAAGGAG
Sut1 (GRMZM2G034302)	Fw: GGCACAAGTGGTTTCCGTTC
	Re: TTTGCCTTTGTGGGGAGGTT
Sus1 (GRMZM2G152908)	Fw: CGTACACCGAGTCGCACAAGAG
	Re: TCCACCAGCCCAGTCAAGTTCT
Ubiqutin	Fw: TAAGCTGCCGATGTGCCTGCGTCG
	Re: CTGAAAGACAGAACATAATGAGCACAG
β-Tubulin	Fw: CTACCTCACGGCATCTGCTATGT
	Re: GTCACACACACTCGACTTCACG

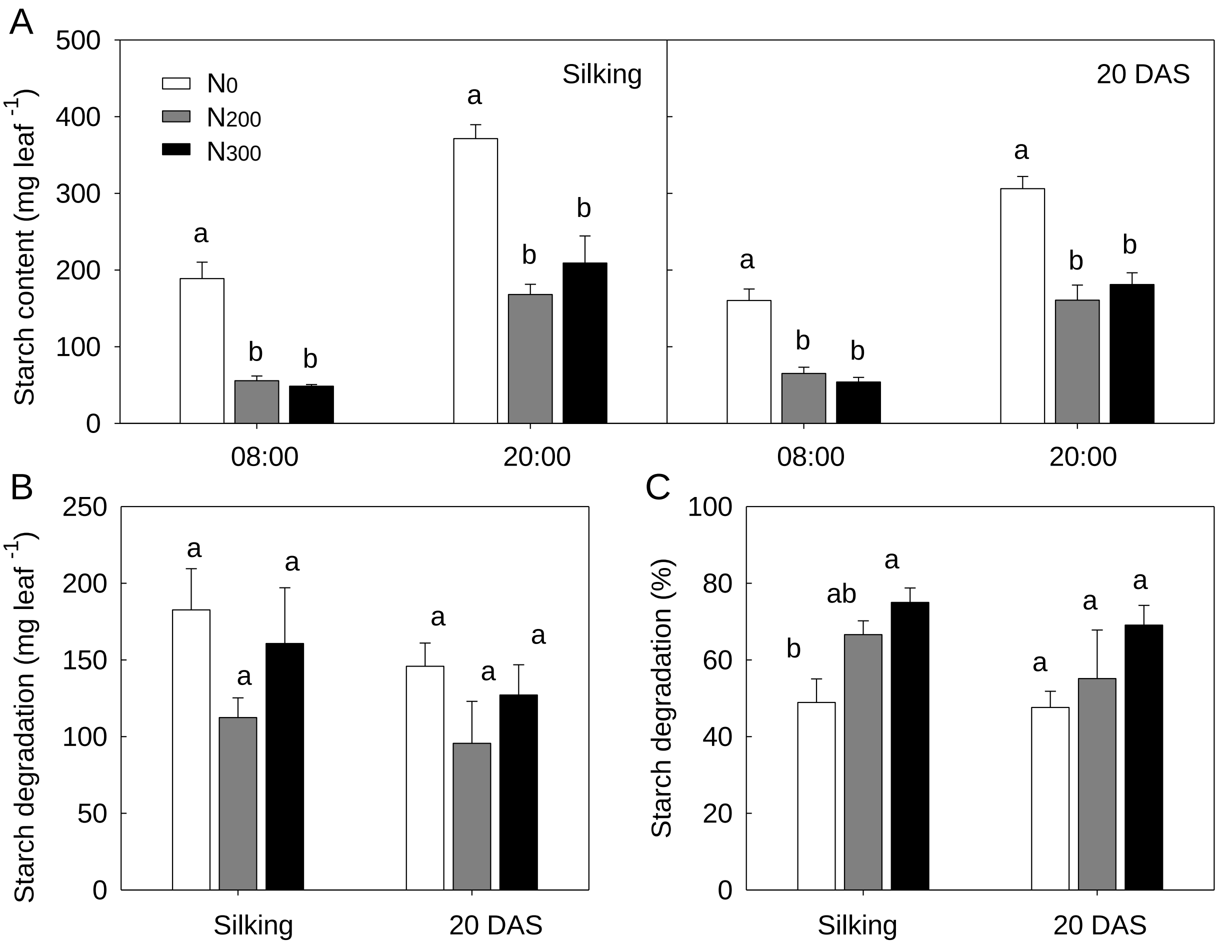

Figure S1. The amounts of starch accumulation and degradation in ear leaves of maize under different nitrogen supply. Samples were collected at 20:00 and 08:00 at each harvest in 2015. Bars represent the standard error of the mean (n=4). Different letters above the columns at each harvest represent the significant differences between nitrogen levels (P<0.05).

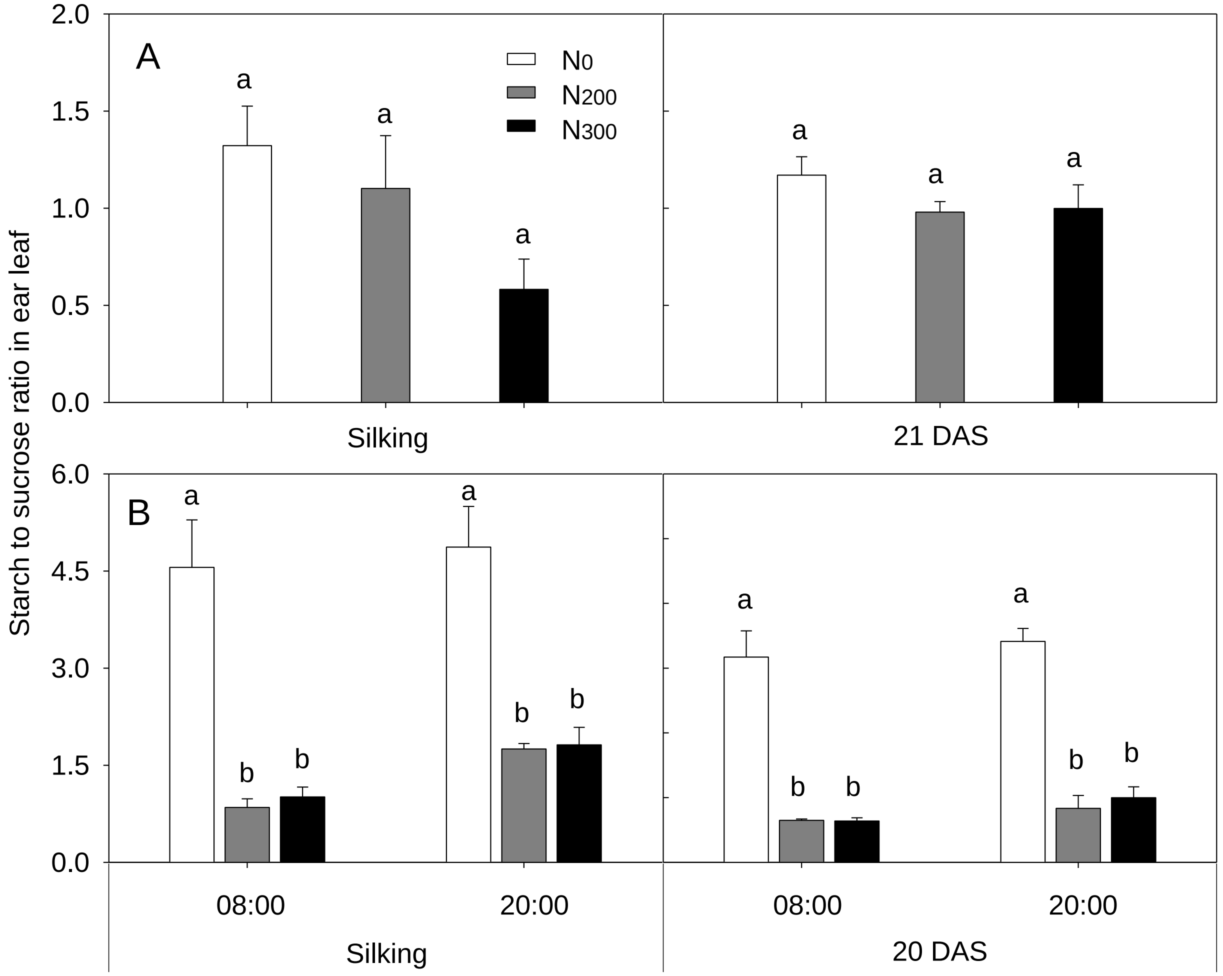

Figure S2. Starch to sucrose ratio in maize ear leaves at silking and 20 or 21 days after silking. Ear leaves were collected between 09:00-11:00 in 2014 (A), and at 08:00 and 20:00 in 2015 (B), respectively. Bars represent the standard error of the mean (*n*=4). Different letters above the columns at each harvest represent the significant differences between nitrogen levels (P<0.05).

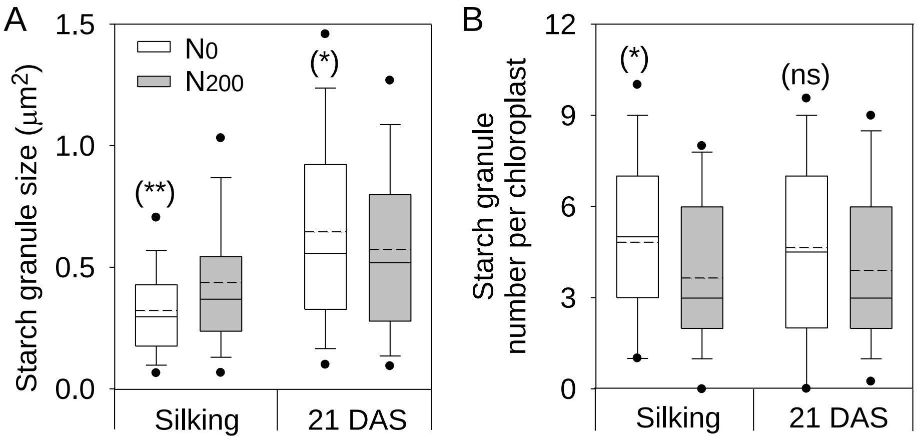

Figure S3. The size (A) and number (B) of starch granules in bundle sheath cells of maize ear leaf at 21 days after silking in 2014. *, P<0.05; **, P<0.01; ns, not significant.
